# The UTH-UMB Global Health Education Collaboration: Building a Bidirectional Exchange Based on Equity and Reciprocity

**DOI:** 10.5334/aogh.3718

**Published:** 2023-08-11

**Authors:** Francis Mupeta, Suilanji Sivile, Mona-Gekanju Toeque, Sombo Fwoloshi, Paul Msanzya Zulu, Duncan Chanda, Nyuma Mbewe, Mundia Mwitumwa, Chitalu Chanda, Nyakulira Kandiwo, Luunga Ziko, Tilele Mwansa, Peter Matibula, Anchidinka Mugala, Edward C. Traver, Ravi K. Tripathi, Emily L. Heil, Devang M. Patel, David J. Riedel, Lottie Hachaambwa, Lloyd Mulenga, Cassidy W. Claassen

**Affiliations:** 1 Division of Infectious Diseases, Department of Medicine, University of Zambia School of Medicine, Lusaka, ZM; 2 Adult Infectious Diseases Center, University Teaching Hospital, Lusaka, ZM; 3 Ministry of Health, Ndeke House, Lusaka, ZM; 4 Center for International Health, Education, and Biosecurity, University of Maryland School of Medicine, Baltimore, MD, USA; 5 University of Maryland School of Medicine, Baltimore, MD, USA; 6 Department of Internal Medicine, Ndola Teaching Hospital, Ndola, ZM; 7 Zambia National Public Health Institute, Lusaka, ZM; 8 University of Maryland School of Pharmacy, Baltimore, Maryland, USA

**Keywords:** Global medical education, Global health, sub-Saharan Africa, LMICs, global health equity, global health decolonization

## Abstract

The global health exchange program between the University Teaching Hospitals (UTH) of Lusaka, Zambia and the University of Maryland, Baltimore (UMB) has been operating since 2015. As trainees and facilitators of this exchange program, we describe our experiences working in Lusaka and Baltimore, and strengths and challenges of the partnership. Since 2015, we have facilitated rotations for 71 UMB trainees, who spent four weeks on the Infectious Disease (ID) team at UTH. Since 2019 with funding from UMB, nine UTH ID trainee physicians spent up to six weeks each rotating on various ID consult services at University of Maryland Medical Center (UMMC). Challenges in global health rotations can include inadequate preparation or inappropriate expectations among high-income country trainees, low-value experiences for low- and middle-income country trainees, lack of appropriate mentorship at sites, and power imbalances in research collaborations. We try to mitigate these issues by ensuring pre-departure and on-site orientation for UMB trainees, cross-cultural mentored experiences for all trainees, and intentional sharing of authorship and credit on scientific collaborations. We present a description of our medical education collaboration as a successful model for building equitable and reciprocal collaborations between low- and middle-income countries and high-income countries, and offer suggestions for future program initiatives to enhance global health education equity among participants and organizations.

## Introduction

Global health exchanges between academic medical institutions offer unique opportunities for trainees to learn from each other and observe medical practice in different contexts. Such exchanges have increased in popularity; over 200 American universities now offer a global health experience [1]. Of 154 allopathic medical schools in the United States, 140 offered international electives between 2016–2017 [2]. International medical experiences whereby learners from high-income countries (HICs) travel to low- and middle-income countries (LMICs) can be inequitable, potentially resulting in exploitation and harm [3]. Traditional colonialism was characterized by exploitation of knowledge, materials, and both natural and human resources from LMICs; in contrast, inequitable medical exchanges may promote neocolonization of global health, characterized by extraction of experiences, education, and research data [4,5]. Institutional relationships rooted in respect, commitment to mutual capacity building, and health outcomes aligned with LMIC priorities are crucial to building equitable global health partnerships [6].

Since 2015, the University Teaching Hospitals (UTH) of Lusaka and the University of Maryland, Baltimore (UMB) have jointly conducted a global health exchange program. In this Viewpoint, authored by UTH and UMB physicians, we survey the current status of global health exchanges, discuss essential characteristics of equitable collaborations, and propose enhancements to improve these exchanges.

## Current Issues in Academic Global Health Exchanges

Many academic global health exchanges encounter comparable obstacles: inappropriate attitudes and competencies of trainees in new contexts, non-relevant training for learners, and imbalanced benefits and equity in research initiatives.

Trainees from HICs often travel to LMICs with minimal understanding of the cultural, medical, and socio-economic environment, potentially resulting in interactions with domineering or colonizing connotations. Trainees may have an appropriate approach to clinical management, but lack contextual understanding: an American doctor in Zambia might insist on a lumbar puncture, not understanding local cultural taboos against spinal procedures [7]. Such experiences highlight the need for pre-travel training and orientation on cross-cultural and societal differences, norms, medical cultures, and learning styles, as well as differences in disease epidemiology and approaches to diagnosis and treatment.

Training experiences provided to LMIC trainees in HICs may offer limited educational value due to mismatches between HIC resources and local environments. HIC training opportunities may also contribute to ‘brain drain’ by providing incentives and opportunities to remain in HICs. Though many LMIC clinicians and researchers wish to stay in their own country [8], some migrate due to limited resources, lack of training and research opportunities in LMICs, or higher employment, salary, and perceived increased quality of life in HICs [9,10]. Educational and training opportunities across LMICs are needed to mitigate the brain drain crisis, while still allowing LMIC trainees to enhance their training and career in HICs [11,12].

Finally, HIC-LMIC joint research has historically been inequitable to LMIC collaborators: LMIC authors are often under-represented on scientific publications, with articles on Africa having only 44% LMIC authorship [13]. Barriers to research and publication in LMICs include lack of funding opportunities, training resources, research management, technology, infrastructure, and access to scientific literature [14,15]. ‘High-impact’ studies are often conducted by HIC researchers in LMICs but rarely led by LMIC researchers [16]. This further causes LMICs to abandon innovation, instead settling for less costly, ‘lower-impact’ studies such as epidemiologic studies and case reports. Even when LMICs produce high impact research, journal publication fees are prohibitively high, further stifling scientific productivity [13].

## The UTH Masters of Medicine in Infectious Diseases

The collaboration between UMB and UTH has a longstanding history and has evolved into an equitable medical education partnership [17]. In 2005, the Zambian Government requested assistance from UMB to develop Zambian capacity to address the growing HIV/AIDS epidemic. Partnering with the Ministry of Health (MOH), UMB provided medical and technical expertise on care and treatment for people living with HIV, developed healthcare workforce capacity, and facilitated medical education [17,18].

The Masters of Medicine in Infectious Diseases (MMed ID) represents a novel ID specialist training program that emphasizes equitable and collaborative models to improve healthcare training [18]. The program began as a Masters of Science in HIV (MSc HIV) in 2009 via a UMB, UTH, University of Zambia, and MOH partnership; in 2014, it then evolved into MMed training and by 2018 fully transitioned to local leadership. Candidates complete five years of postgraduate training in ID and internal medicine. The program is led by Zambian MMed ID physicians, with support from two Zambia-based UMB ID faculty physicians and a UMB Global Health Fellow. These faculty support ID training and education at UTH via clinical teaching rounds, didactics, and research, as well as the UTH rotation at UMMC.

The MMed ID program contributes to global health equity by transferring skills and developing local training capacity. MMed ID graduates have the training to practice in any global setting while continuing to develop a cadre of health professionals. The MSc HIV/MMed ID programs have graduated over 41 doctors, with over 90% of these physicians taking up clinical and public health leadership positions in Zambia [18].

## The UTH/UMB Global Health Exchange Program

To complement opportunities in the MMed ID program, UMB and UTH trainees have participated in bidirectional exchanges since 2015. UMB trainees, including medical students, residents, pharmacy trainees, and ID fellows, visit UTH for a four-week structured rotation. Trainees round with the inpatient ID consultation service and see patients at the HIV clinic. All rotations are supervised by a Zambian MMed ID senior trainee or graduate and UMB faculty. UMB trainees participate in medical education with UTH MMed ID registrars and contribute to didactic sessions by presenting lectures or case conferences. Seventy-one UMB trainees have rotated at UTH since 2015.

UTH MMed ID registrars spend up to six weeks rotating at the University of Maryland Medical Center (UMMC) in Baltimore. The MMed ID registrars round with UMMC consultation services in carefully selected ID sub-specialties not available in Zambia, such as transplant, surgical, and oncology ID, which both reinforce core ID concepts and enhance understanding of novel diagnostic and therapeutic modalities. MMed ID trainees attend didactic sessions, including core curriculum lectures, case presentations at ID grand rounds, microbiology rounds, journal clubs, and morbidity and mortality conferences. At the end of the rotation, they present a case at both the UMMC ID and UTH ID conferences.

Medical rotators from HICs working in LMICs sometimes participate in procedures beyond their level of training [19]. For parity and equity, we follow similar expectations at both sites: trainees are observers, they participate in rounds, engage in patient interviews, conduct physical exams under supervision, and can review the files and discuss the patient, but do not take primary histories or perform procedures.

The UTH-UMB exchange has created opportunities for both institutions to partner in research. Over the past eight years, the collaboration published 10 abstracts at international scientific conferences, nine peer-reviewed manuscripts, three more manuscripts under review, as well as clinical antibiotic stewardship guidelines for UTH [20]. All publications include trainees and faculty from both institutions. The UTH-UMB ID team also serves as a key technical resource for the MOH and contributes to national guidelines and policy for HIV [21], PrEP [22], antibiotics [20], tuberculosis [23], and COVID-19 [24].

For the first three years, the collaboration did not have independent funding and trainees paid their own way or applied for small grants. Since 2019, the University of Maryland School of Medicine, via the Center for International Health, Education, and Biosecurity (Ciheb) at the Institute of Human Virology and the President’s Global Impact Fund (PGIF), via the UMB Center for Global Engagement (CGE), supported the UTH-UMB Global Health Collaboration. Funds are equally applied to each institution; to date, nine UTH and 15 UMB trainees have been supported. It is more costly for Zambians to visit Baltimore, hence the discrepancy in numbers.

## Pedagogical Elements of the UTH/UMB Global Health Bidirectional Exchange

The rotations at UMB and UTH include a co-developed curriculum that covers medical knowledge, practices, and culture encompassing Eichbaum’s paradigm shift of global health: patient safety, fair trade, co-developed curricula, power dynamics, and equalization of access and opportunity [4]. Using this framework, we attempt to intentionally address HIC trainee preparedness and mentorship, LMIC trainee value, and equity in collaborative research.

To ensure UMB trainees are prepared for their time in Zambia, all aspiring participants meet with UMB faculty in Baltimore prior to acceptance. We discourage participation for the purposes of resume enhancement, interview talking points, or simply vacation, which have all been cited as motivations for global health rotations [25]. Upon arrival, trainees receive orientation from UMB faculty regarding Zambian history, culture, and medical norms, including respectful patient and interprofessional interactions. These lessons enable successful clinical learning while at UTH, improve acceptability among Zambian patients and healthcare providers, and mitigate potentially destructive properties of neocolonial global health exchanges.

The orientation invokes Abimbola’s ‘principle of subsidiarity’ in global health: proximate actors at ground level hold the most knowledge about their domain and decisions should take place at that level, i.e., ‘default to the local gaze,’ with ensuing practical and moral benefits [26]. UMB trainees understand that they are to learn from Zambian healthcare practitioners (not vice versa), as the Zambians are ‘experts who are closest to the problems… and closest to the solutions’ [27].

The UMMC rotation for Zambian MMed ID registrars strives to be beneficial and impactful. While in Baltimore, MMed trainees are mentored by UMB ID faculty that work in Zambia or other sub-Saharan African countries; this creates a shared baseline for focused and impactful learning. The UMB rotation is timed to coincide with UMB Zambia-based faculty attending at UMMC, to preserve continuity of mentorship and education. Infectious disease rotations are structured to reinforce key concepts in ID (i.e., principles of antibiotic therapy, antibiotic stewardship, and microbiology) that strengthen the trainees’ ID expertise. Finally, the UMB rotation is used to launch collaborative research, with connections to mentors and projects. By providing exchange opportunities and education for MMed trainees, we aim to develop the next cadre of doctors to provide ID medical care at the highest standards in Zambia.

To build equity in scientific collaboration, we developed a shared authorship model for collaborative research projects. In doing so, we aim to mitigate against parachute or parasitic research [28], imbalanced power dynamics [4,6], and to ‘amplify the voices of the health workers and researchers in LMICs for whom the notion of “global health” is but an everyday reality of their working lives’ [16]. All collaborative UTH-UMB manuscripts have either a UTH first or last author, to ensure that ‘local people [are] writing about local issues for a local audience’ [26]. In the 11 manuscripts published or submitted between 2015 and 2022, all have at least three UTH-affiliated authors, 10 have a UTH-affiliated last author, and six have a UTH-affiliated first author.

## Challenges Encountered

Developing this collaboration is not without challenges, including ongoing and sustainable funding, students’ expectations, bureaucratic hurdles for LMIC rotators, co-developing research, and pandemic travel restrictions.

The greatest challenge faced by the collaboration is funding. Initially, UMB trainees paid their own way or were supported by independent scholarships; there was no support for Zambians to travel to Maryland. Because trainees were self-funded, this inherently selected for people of means with a potential sense of entitlement. Until 2018, the two Zambia-based UMB faculty were funded in part via PEPFAR to support medical education; when this grant ended, they continued their work *pro bono* and developed a funding proposal for UMB. In 2019, Ciheb and CGE provided internal funding for the UTH-UMB collaboration, which supports travel and lodging for all trainees, small stipends for Zambians while in Baltimore, and salary support for the Zambia-based UMB faculty. The funding proved truly transformative: by confronting resource gaps, we made significant progress towards decolonization and building equitable relationships.

Another hurdle is the extensive bureaucratic requirements faced by Zambian trainees when coming to UMMC, which American trainees do not face going to UTH; these include USA visa applications and extensive hospital paperwork. The hospital rules for observers at UMMC also limit access to electronic records and hands-on physical examination of patients. The cost of living is substantially higher in the USA than in Zambia, placing an economic burden on visiting trainees that is only partially offset by a small stipend.

Lack of pre-established understanding on authorship distribution caused challenges during our first forays into collaborative research. Collaborators from UTH and UMB had to have difficult conversations to determine author order and credit. Pre-existing relationships built on mutual trust and respect enabled successful discussions that resulted in our current model of shared authorship.

The COVID-19 pandemic imposed hurdles due to international travel restrictions. When exchanges were paused in April 2020, we began weekly virtual case discussions and journal clubs, which maintained both academic learning and professional relationships. Following the lifting of travel restrictions in May 2022, the exchanges are now back in full effect.

## Equity and Decolonization via Global Health Exchanges: Future Opportunities

As global health physicians, we believe that global health equity can be fostered by promoting LMIC science, developing intersectional approaches, and decreasing knowledge and resource gaps between LMICs and HICs.

Scientific ideas from LMICs in research, clinical management, and innovation should be brought to scale and visibility. Unstated standards or norms in global health education govern transfer of ideas generated in the HICs to LMICs; science is often produced with ‘the foreign gaze’ in mind [26]. To develop equity in global health education, we need a paradigm shift so that impactful innovations generated from LMICs can play a key role in HICs [4,6]. Low- and middle-income countries need support for development of local innovations, as opposed to imported solutions from HICs, which are often unsustainable due to financial limitations.

Intersectionality assesses relationships and interactions between factors rather than examining individual factors themselves; it allows for assessment at multiple levels in society to determine how health is shaped across population groups and geographical context [29]. Using an intersectional approach to global health highlights differences within populations and advances a systematic approach to understanding inequalities in global health [29,30].

One of the great inequalities in medical education and global health is the lack of access to resources and tools available for fostering research in LMICs. As data becomes more available, resources to strengthen research output would improve scientific productivity for LMIC researchers. Measures to decolonize medical education and global health should be redesigned to consciously focus on education and provide access to research skills.

## Conclusion

The UTH-UMB global health collaboration is intended to promote reciprocal exchange with commitment to equitable training and professional development. The structure of these rotations enhances healthcare capacity building, transfer of knowledge, and contextual cultural learning to advance global health equity. Successful elements of our program include on-site culturally-competent mentorship, maximizing education for the learners’ context, and collaborative research.

Building global health equity requires developing medical education aligned with the needs of LMICs. We advocate for global health exchanges with targeted education centered on perspectives of medical education and cultures, including dialogue around neocolonialism and decolonization of global health. Future aspirations include bridging the gaps in medical education and promoting equity via increased access to technology and long-term educational opportunities with mentorship between HIC and LMIC trainees.

There are significant and complex barriers to these proposed exchanges, including resource limitations. We propose that the UTH-UMB collaboration can serve as an example of global health equity beyond addressing basic resource limitations, while acknowledging that continued dialogue around intersectionality and neocolonialism is necessary and ongoing.
